# Expanding phenological insights: automated phenostage annotation with community science plant images

**DOI:** 10.1007/s00484-025-02972-x

**Published:** 2025-07-04

**Authors:** Negin Katal, Michael Rzanny, Patrick Mäder, David Boho, Hans Christian Wittich, Susanne Tautenhahn, Anke Bebber, Jana Wäldchen

**Affiliations:** 1 Department Biogeochemical Integration, Max Planck Institute for Biogeochemistry, Hans-Knöll-Str.10, 07745 Jena, Thuringia Germany; 2 Faculty of Biological Science, Friedrich Schiller University, Fürstengraben 1, 07743 Jena, Thuringia Germany; 3 Data Intensive Systems and Visualisation, Technische Universität Ilmenau, Ehrenbergstraße 29, 98693 Ilmenau, Thuringia Germany; 4 German Centre for Integrative Biodiversity Research (iDiv), Halle-Jena-Leipzig, Leipzig, Germany

**Keywords:** Opportunistic plant observations, Phenology, Citizen science, Community science, DWD, Machine learning, Phenostage

## Abstract

Plant phenology plays a pivotal role in understanding the interactions between plants and their environment. Despite increasing interest in plant phenology research, documenting their spatial and temporal variability at large spatial scales remains a challenge for many species and a variety of phenostages. The use of plant identification apps results in a vast repository of plant occurrence records spanning large spatial and temporal scales. As these observations are usually accompanied by images, they could potentially be a rich source of fine-grained large scale phenological information. However, manually annotating phenological stages is time intensive, necessitating efficient automated approaches. In this study, we developed a machine learning-based workflow to automatically classify plant images into the phenological stages of flowering bud, flower, unripe fruit, ripe fruit, and senescence for nine common woody shrub and tree species. Although the process required only a small amount of training images, the classification achieved an overall accuracy of 96% across all species and phenostages. To evaluate the phenological relevance of these automatically annotated observations, we compared their temporal and spatial patterns from three years (2020–2022) with systematically collected phenological data from the German Meteorological Service (DWD). Our results revealed strong spatial and temporal consistency, particularly for the flowering stages, with interannual phenological trends aligning well between the datasets. Our results demonstrate that automatic annotation of phenological stages can be achieved with high reliability even with low manual labeling effort. Provided that a high number of images is available, these automatically labeled observations carry a strong phenological signal.

**Supplementary Information:**

The online version contains supplementary material available at 10.1007/s00484-025-02972-x.

## Introduction

Plant phenology, the study of the timing of recurring life events such as budburst, flowering, fruiting, and senescence, is critical for various ecological processes. It influences individual plant fitness, population dynamics, plant-animal interactions, and key ecosystem processes, including carbon sequestration and nutrient cycling (Richardson and O’Keefe 2009; Cleland et al. 2007). Therefore, continuous monitoring, evaluation, and modeling of phenological patterns are essential for understanding plant responses to environmental changes and for assessing the cascading effects on broader ecosystem functions and processes (Noormets 2009).

Numerous phenological networks worldwide systematically document phenological events at the level of individual species (e.g.: European Phenology Network (EPN) (van Vliet et al. 2003), International Phenological Garden Network (IPG) (Renner and Chmielewski 2022), PhenObs (Nordt et al. 2021). National institutions also manage phenological monitoring programs, engaging community scientists to record phenological changes over large regions (Beaubien and Hamann 2011; Taylor et al. 2019). Observation networks run by national institutions often focus on a limited set of taxa and and sites, resulting in spatially sparse data sets (Puchałka et al. 2022). In Germany, the German Meteorological Service (Deutscher Wetterdienst, DWD) maintains one of the most extensive networks, collecting long-term phenological data of several species with thousands of trained volunteers who follow well-defined protocols (Kaspar et al. 2015). However, the number of volunteers continues to decline, posing a significant risk to data accuracy and coverage (Yuan et al. 2021).

While systematic phenological records are declining or facing financial challenges (Renner and Chmielewski 2022), a new data stream of opportunistic and unsystematic plant observations is emerging, driven by AI-based plant identification apps (Mäder et al. 2021), already achieving human-level identification accuracy (Wäldchen and Mäder 2018; Jones 2020; Villon et al. 2020; Pärtel et al. 2021; Rzanny et al. 2024a). These technologies allow anyone, regardless of their botanical knowledge, to identify plant species in the field. As a result, large-scale geolocated and time-stamped observations are being collected and provide valuable data for various ecological monitoring tasks (Bonnet et al. 2020; Mahecha et al. 2021). However, despite the popularity of these apps, only a few studies have evaluated their potential for phenological monitoring (Katal et al. 2023; Rzanny et al. 2024b; Mora et al. 2024). Katal et al. (2023) demonstrated that opportunistic plant observations can be used to estimate the onset of flowering by analyzing the observation frequency over time. By using plant observation data collected via the app Flora Incognita, the authors were able to estimate the onset of flowering dates for 11 species, achieving results that were consistent with the systematic records collected by the DWD. Furthermore, a study on a European scale showed that the timing of opportunistic plant observations shifts in response to spatial and temporal gradients (Rzanny et al. 2024b). These studies demonstrate that for species characterized by a single conspicuous phenological event, such as flowering, the observation density curve over time can be directly related to that event, i.e. flowering time.

For species with no or multiple conspicuous phenological stages, the resulting observation curves will show no or multiple distinct peaks throughout the year, rendering this methodological approach as not applicable.

Observations from identification apps are accompanied by an image of the identified plant, enabling a manual phenostage assignment (Barve et al. 2020; Puchałka et al. 2022; Klinger et al. 2023). However, this process is highly labor-intensive, as community science datasets often contain thousands of images (Klinger et al. 2023; Barve et al. 2020; Puchałka et al. 2022). Convolutional neural networks (CNNs) have been successfully applied to the automatic classification of phenological stages across various settings, including remote sensing, herbarium specimens, and community science data (Katal et al. 2022). However, these models are computationally demanding, often requiring thousands of images for effective training. Efficient methods with minimal data and computational needs are crucial for expanding phenological research applications.

One effective approach is feature transfer, which uses a pre-trained CNN to extract key information from images by converting them into numerical feature representations (known as deep features). These features significantly reduce the image’s complexity while preserving relevant patterns. The extracted deep features are then used to train a simpler model, such as a Support Vector Machine (SVM), which is widely used for classification and regression tasks. This method is computationally efficient, less prone to overfitting, and performs well even with small, unbalanced datasets (Donahue et al. 2014; Oquab et al. 2014; Xiao et al. 2019). Recent studies have demonstrated its effectiveness in image classification with limited labeled data, e.g. the automated recognition of Poaceae species (Rzanny et al. 2022).

Here, we present a workflow for developing an automated phenological stage classifier that utilizes deep feature extraction from iNaturalist images, combined with an SVM classifier. This approach significantly reduces the need for large training datasets. We apply the model to over 600,000 plant images collected by the Flora Incognita app, to automatically annotate the observations with specific phenological stages. To validate our results, we compared the spatial and temporal variations of the automatically annotated phenological stages from these opportunistic plant observations with systematic phenological observations from the DWD, thereby assessing the potential of this approach for phenological research.

## Materials and methods

**Fig. 1:**
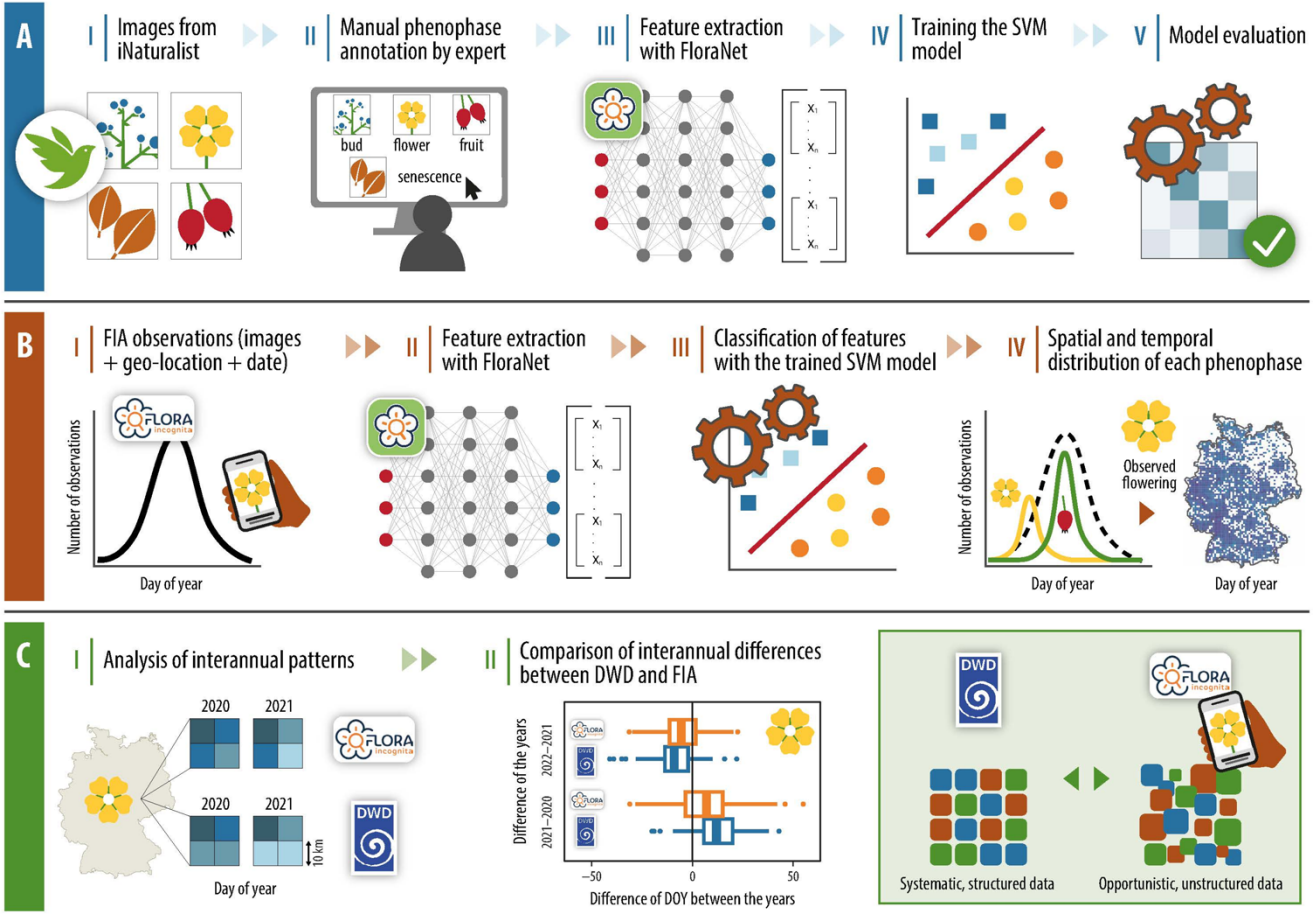
The workflow of this study can be separated into three main steps. **A**) Development of a phenostage classifier. Species- and phenostage-annotated plant images from *iNaturalist* were fed to FloraNet for feature extraction. The resulting features were used to train a Support Vector Machine classifier discriminating all 39 phenostages listed in Table 1. **B**) Annotating phenostages on images collected via a plant ID app and estimating their median observation date Plant observations collected by the plant ID app *Flora Incognita* (FIA) were classified as specific phenostages using the classifier developed in step A). These observations are used to model the onset of a particular phenostage on a 10 × 10 km grid across Germany. **C**) Comparing the spatiotemporal changes in phenology to reference observations. The temporal occurrence of each phenostage was compared to the observations from the German Meteorological Service’s (DWD) annual phenology monitoring reports

Our approach consists of three main steps, which are summarized in Fig. 1. First, we train a phenostage annotation model using plant images from *iNaturalist*, followed by the evaluation of its performance (Section"Classifier development"). Second, we use the trained model to automatically annotate phenostages on images of plant observations collected by the plant identification app Flora Incognita (FIA) (Section"Phenostage classification"). Finally, we conduct a case study using phenological observation data from the DWD to assess whether the interannual differences in the timing of the automatically annotated phenological stages are consistent with the timing observed and documented in the DWD’s annual phenological reports within Germany (Section"Comparison to reference data").

### Classifier development

#### Species selection and training data set

In order to facilitate a comparison with reference data, species selection was based on three criteria: (a) they are monitored by the DWD, (b) with at least two phenostages, and (c) they should be frequently (more than 1000 observation per year) recorded via the FIA. A total of nine plant species met these criteria, as shown in Table S1: four tree species: *Acer platanoides*, *Aesculus hippocastanum*, *Betula pendula*, *Fagus sylvatica* and five deciduous shrubs or small trees species *Cornus mas*, *Crataegus laevigata*, *Rosa canina*, *Sambucus nigra*, *Sorbus aucuparia*.

Training images for building the phenostages annotation classifier were obtained from the iNaturalist platform (https://www.inaturalist.org/). The image selection process was based on iNaturalist phenostage categories and their location (Europe). The phenostage categories were:”flower”,”fruit or seeds”,”flower buds”,”no flower or fruits”, and”any”. The images were manually reviewed to verify that the phenostage matched the target category of interest.

The resulting number of images per phenostage varied highly, depending on availability; ranging from 20 for flowering of *Betula pendula* to 132 for flowering of *Rosa canina*. In total, 2,890 images were downloaded for the nine selected species.

The images were manually categorized into stages “*seedling*”, “*flowering bud*”, “*flowering*”, “*fruit*”, “*unripe fruit*”, “*ripe fruit*”, “*senescence*” and “*vegetative*”. The “*seedling*” stage was recorded exclusively for *Fagus sylvatica* and assigned when newly germinated plants, characterized by small cotyledons or emerging primary leaves, were present. The “*flowering bud*” stage was assigned when one or more unopened flower buds were visible. The “*flowering*” was assigned to images where at least one open flower was visible. The “*fruit*” stage was divided into two categories: “*unripe fruit*” when fully formed but green fruits appeared without remaining flower petals, and “*ripe fruit*”, when fruits reached maturity and showed coloration such as red, brown, or black, indicating their ripening. For *Fagus sylvatica*, *Acer platanoides* and *Betula pendula*, the distinction between unripe and ripe fruit was difficult due to the gradual transition between these stages and the fact that the fruits were often photographed from a distance. So, we did not classify the fruits into the two distinct stages, but instead assigned them only to the fruit stage. The “*senescence*” stage was assigned when yellow, orange, or red leaves were visible. If no reproductive organs were visible, the images were classified as “*vegetative*”, which also included brown and dry leaves. The phenostages were treated as discrete categories; images containing multiple reproductive stages were labeled according to the most advanced phenostage (e.g., images showing both flowering and fruiting were classified as fruiting). An overview of image examples and the number of labeled images per stage and species is provided in Table S1.

#### Feature extraction, training and evaluation

The development of the phenostage classifier involved two subsequent steps: feature extraction using a pre-trained neural network, followed by the training of an SVM classifier using the extracted feature vectors (see Fig. 1 panel A III and IV).

The pre-trained deep neural network used for feature extraction is”*FloraNet*” (Mäder et al. 2021). During the feature extraction stage, high-dimensional image data are projected into a lower-dimensional feature space, representing each image as a vector with the length of 1792 elements. The feature vectors where then used to train an SVM (Cortes and Vapnik 1995) with 39 classes to classify the extracted features to all species-phenostage combinations. SVMs have been shown to achieve higher accuracies compared to other algorithms in similar classification tasks (Rzanny et al. 2022) and have been successfully applied (Valan et al. 2019; Rzanny et al. 2022; Hodač et al. 2024) due to their effectiveness in high-dimensional spaces and their ability to handle small and unbalanced data sets well (He and Garcia 2009). The data set was divided into subsets for training and testing, with 80% of the data allocated to training and 20% reserved for testing (see Table 1). The model was evaluated using overall accuracy, which measures the proportion of correctly classified phenostages, providing a general assessment of predictive performance (Grandini et al. 2020).

**Table 1:** Classification performance metrics of the Support Vector Machine (SVM) model, along with the number of images per class and species used for training and evaluation

Species	Phenostage	Number of Training images	Number of Tes images	Identification performance		
				F1	Recall	Precision
	flower	80	19	1	1	1
*Acer*	fruit	73	18	1	1	1
*platanoides*	senescence	57	14	0.96	0.92	1
	vegetative	84	20	0.97	1	0.95
	flowering bud	33	8	0.82	0.87	0.77
*Aesculus*	flower	64	15	0.93	0.93	0.93
*hippocastanum*	unripe fruit	64	15	0.96	1	0.93
	ripe fruit	64	15	1	1	1
	vegetative	64	15	0.92	0.86	1
	flower	32	7	0.93	1	0.87
*Betula*	fruit	16	4	1	1	1
*pendula*	senescence	32	7	0.83	0.71	1
	vegetative	31	7	0.80	0.85	0.75
	flower	65	16	1	1	1
*Cornus mas*	unripe fruit ripe fruit	39 77	9 19	0.88 0.97	0.88 0.94	0.88 1
	vegetative	44	10	0.90	1	0.83
	flowering bud	32	6	0.88	0.87	0.87
*Crataegus*	flower	50	12	0.95	0.91	1
*laevigata*	ripe fruit	46	11	1	1	1
	vegetative	52	13	0.92	0.92	0.92
	seedling	19	4	1	1	1
	flower	19	4	1	1	1
*Fagus sylvatica*	fruit	79	19	1	1	1
	senescence	90	22	0.95	0.95	0.95
	vegetative	54	13	0.92	0.92	0.92
	flower	106	26	1	1	1
*Rosa canina*	ripe fruit	80	19	1	1	1
	vegetative	74	18	1	1	1
	flowering bud	78	19	0.94	0.94	0.94
*Sambucus nigra*	flower unripe fruit ripe fruit	86 80 97	21 20 24	1 0.90 0.95	1 0.90 0.95	1 0.90 0.95
	vegetative	68	16	1	1	1
	flowering bud	32	7	0.93	1	0.87
*Sorbus aucuparia*	flower ripe fruit senescence	86 84 19	21 20 4	0.97 0.95 0.66	0.95 1 0.50	1 0.90 1
	vegetative	81	20	1	1	1
Overall accuracy: 96%						

To evaluate the performance for individual phenostages and species, we calculated Precision, Recall, and F1 score (Grandini et al. 2020). Precision indicates the proportion of correctly identified instances among those predicted as a given class, while Recall reflects the proportion of correctly identified instances among all true instances of that class. The F1 score represents the harmonic mean of Precision and Recall.

### Phenostage classification

#### Opportunistic plant observation data

Opportunistic plant observations were obtained from Flora Incognita, a plant identification app designed to reliably identify plant species, made for both botanical novices and experts (Mäder et al. 2021). Users capture and identify plants based on their personal interests, without being guided to observe specific species or phenological stages. Consequently, the resulting observations can be classified as opportunistic, as they arise spontaneously and reflect individual user preferences rather than systematic or standardized monitoring efforts.

For this study, we utilized observations of the nine target species collected between January 1, 2020, and December 31, 2022 yielding a total of 627,885 observations. Each includes an image, geolocation, and the date of recording. An overall overview of the data set is provided in Table S2.

#### Processing of the plant observation data

The image features were extracted via “FloraNet”, consistent with the methodology applied to iNaturalist images (see Section"Feature extraction, training and evaluation"). The extracted features were then subjected to the trained SVM classifier, which assigned each image to one of the 39 species-phenostage combinations. Each FIA observation was then annotated with the resulting phenostage label (see Fig. 1 panel B). Afterwards, we identified and removed outliers. These are data points falling outside a meaningful day of year (DOY) time window for the occurrence of a specific phenostage. We used the”Intra-cluster outlier detection” method described by Mehdipoor et al. (2015). Outliers were defined as observations lying beyond 1.5 times the interquartile range (IQR) calculated from the first quartile (Q1) to the third quartile (Q3) (Mehdipoor et al. 2015). This approach was applied separately for each species-phenostage combination and year to exclude outliers within the annual data set for a given phenostage.

Furthermore, we excluded the phenological stage we categorized as"vegetative"from further analysis, as it broadly encompasses all non-reproductive phases in a plant’s life cycle and does not represent a distinct, well-defined phenophase relevant to the specific focus of our study.

### Comparison to reference data

To evaluate the timing of the automatically annotated phenostages with ground truth data, we compared spatiotemporal differences of each phenostage-annotated image with systematic phenological observations provided by the DWD (see Fig. 1, panel C).

#### Systematic phenological plant observation data

The phenological observations of the DWD provide the systematic comparative data used in this study. The DWD oversees a network of trained volunteers and experts who monitor specific plant species throughout the year according to a rigorous protocol. (Zimmermann and Polte-Rudolf 2013; Kaspar et al. 2015). We extracted data from the DWD phenological observation data set (https://opendata.dwd.de/climate environment/CDC/observations germany/phenology/annual reporters/wild/) for all nine species, from January 1, 2020, to December 31, 2022. We focused on phenostages that could be reasonably aligned with opportunistic image observations: flowering onset, leaf senescence, and first ripe fruit; resulting in 39,992 records suitable for this study (Table S3).

#### Comparing spatiotemporal differences

To assess the validity of the phenostage annotation and to demonstrate their use as phenological indicators we performed a grid-based comparison of the phenostages derived from the annotated FIA images to the systematic phenological observations performed by the DWD.

The onset of a systematically captured phenophase refers to the date when the first individual showing of that stage is observed at different locations (Table S3). In contrast, the first opportunistic observation of a phenostage does not necessarily represent its onset. Instead, the capturing of a certain phenostage by chance is strongly dependent on the overall observation count, resulting in a bias towards population-dense locations (Mahecha et al. 2021). To mitigate such sampling intensity effects, it is generally recommended to report mean data instead of onset dates (Moussus et al. 2010; de Keyzer et al. 2017; Jones and Daehler 2018; Iwanycki Ahlstrand et al. 2022). As a consequence, these values measure different progressions of the respective phenostages:”onset” by DWD and”median observation date” by FIA are not directly comparable, for the median observation date will always lag behind the systematic observation. However, the concordance of the estimates would still be comparable between years. This is why we compared the differences in DOY estimates of the same locations in two consecutive years (2020—2021 and 2021—2022) across both data sets instead of comparing the values directly. The more the annual differences are corresponding, the more similar we expect the distribution of estimates to be.

We divided Germany into 5,742 grid cells of 10 × 10 km each. This size is a compromise between resolution and the number of available observations per grid cell. For both opportunistic and systematic data sets and for each species and phenostage across all three years, the median observation date was calculated in grid cells with multiple observations to represent the DOY of that phenostage. For grid cells with only a single observation, the DOY of that observation was used. For each species-specific phenostage and in both data sets, grid cells with observations across all three years were identified and the differences were compared to evaluate consistency and detect potential discrepancies in phenological trends.

We used simple Mantel tests (Mantel 1967) to quantify the correspondence of the DOYs derived from DWD and FIA to test whether the DOYs per phenostage from DWD and FIA were spatially consistent. We calculated an Euclidean distance matrix for each phenostage, species, and year combination and computed the Mantel test statistic (r M) as a measure of correlation between these distance matrices. For each pairwise comparison, we tested the statistical significance of the Mantel statistic using a one-tailed permutation test with 9,999 permutations, randomly shuffling the grid cell values to create a null distribution.

We computed all calculations using R 4.3.2 (R Core Team 2023) version and package *ecodist* for Mantel test (Goslee and Urban 2007) packages *caret* (Kuhn 2008) and *01071* for SVM model (Meyer et al. 2019).

## Results

### Model performance on automated phenostage annotation

Our SVM model trained to classify 39 species-specific phenostages across the nine species achieved an overall accuracy of 96%, correctly classifying 537 out of 559 test images into their appropriate species-specific phenostages. A summary of the evaluation results is presented in Table 1. The F1 scores per class were above 0.9 for 32 of the 39 phenostages. The F1 score of five phenostages ranges between 0.8 and 0.9. Notably, the senescence stage of *Sorbus aucuparia* exhibited the lowest F1 score, reflecting a higher degree of misclassification for this particular phenostage of this species. Fifteen of the 39 phenostages achieved a perfect F1 score of 1.0 (Figure S3). The confusion matrix (Figure S2) reveals that misclassifications occurred primarily between phenostages within the same species. The only instance of an interspecies confusion occurred when a vegetative image of *Cornus mas* was incorrectly classified as a vegetative stage of *Crataegus laevigata*. The F1 scores indicate that the highest identification accuracy is achieved for the flower, followed closely by the flowering bud stage. The unripe fruit stage shows lower accuracy compared to the ripe fruit stage. Among all phenostages, senescence exhibits the lowest F1 score (Figure S3).

Overall, the model, along with the F1 scores per class, exhibits a high degree of accuracy, rendering it suitable for subsequent application to unseen images from FIA.

### Automated phenostage annotation of FIA images

#### Proportion of observations per phenostage for each species

The vegetative stage represented 40–75% of all phenostages in the FIA images across all species (Figure S4) and was excluded from further analyses (342,161 data points). The remaining 285,724 data points represented images that could be assigned to one of the phenostages. Across species, the proportion of flowering and fruiting stages are more or less similar. However, for *Sorbus aucuparia*, fruiting is observed six times more often than flowering. The proportion of flowering bud stage remains consistent across the four species for which it was recorded.

#### Temporal distribution of phenostages per species

Following the outlier removal process described in Section"Processing of the plant observation data", 42,114 observations were removed. After this, the final data set consisted of 243,610 observations, each assigned to a specific phenostage.

**Fig. 2:**
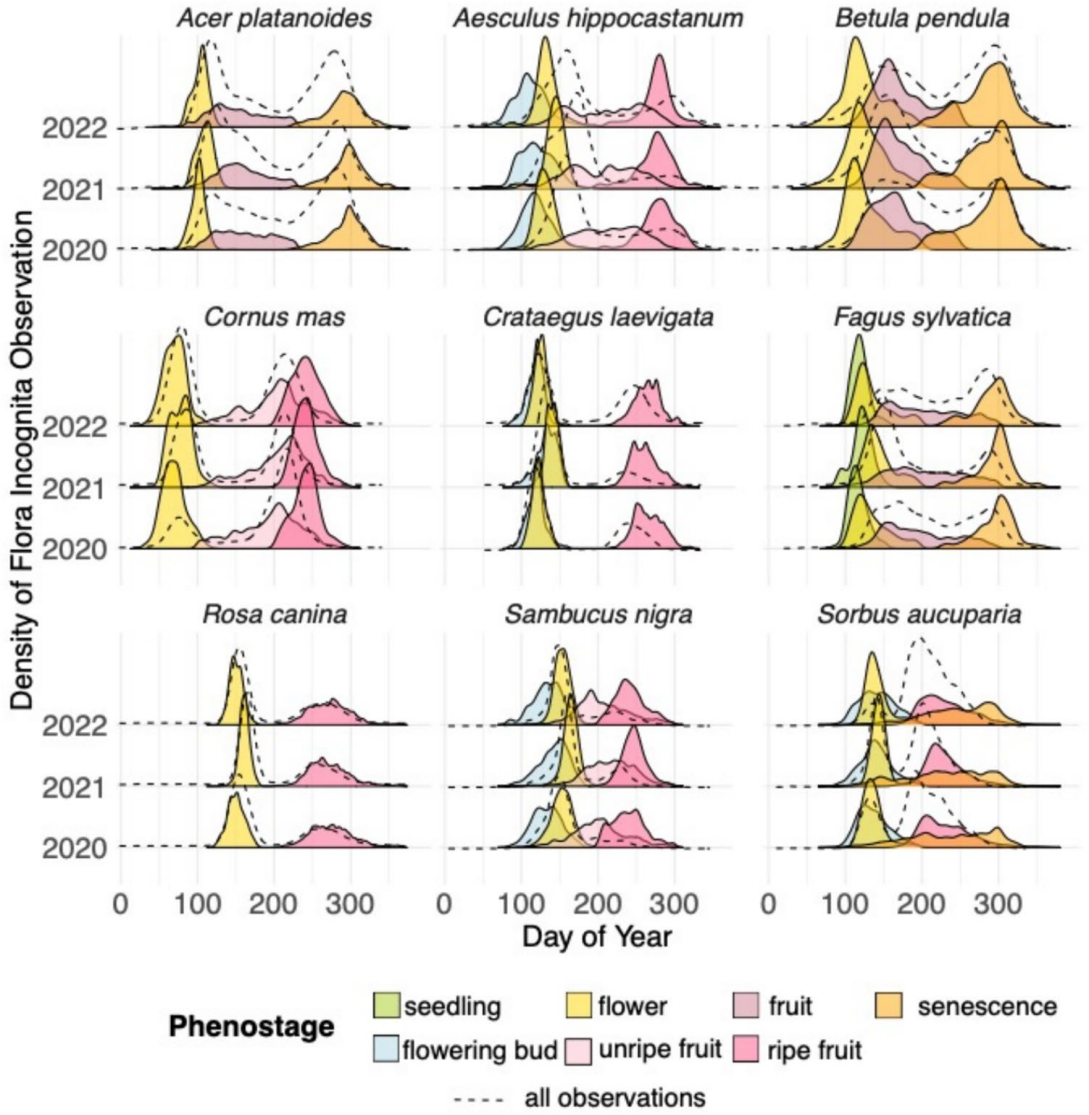
Temporal distribution of each phenostage after automated annotation and outlier removal. The dashed line represents the overall distribution curve before the automated annotation process

To illustrate the temporal distribution of each phenostage throughout the year and assess data consistency across years, we plotted the density of observations per phenostage, species and year as shown in Fig. 2. The specific observation curves per phenostage are well separated and follow the expected temporal progression throughout the year, beginning with the flowering bud, followed by flowering, then fruiting (unripe and ripe fruit) and concluding with senescence. This pattern indicates that the automated phenostage annotation on unseen images from FIA is generally effective. Differences across years are clearly visible in the observation curves, with distinct variations in the timing of the occurrences of phenostages.

### Comparison of systematic phenological records and opportunistic plant observations

#### Quantitative comparison

**Fig. 3:**
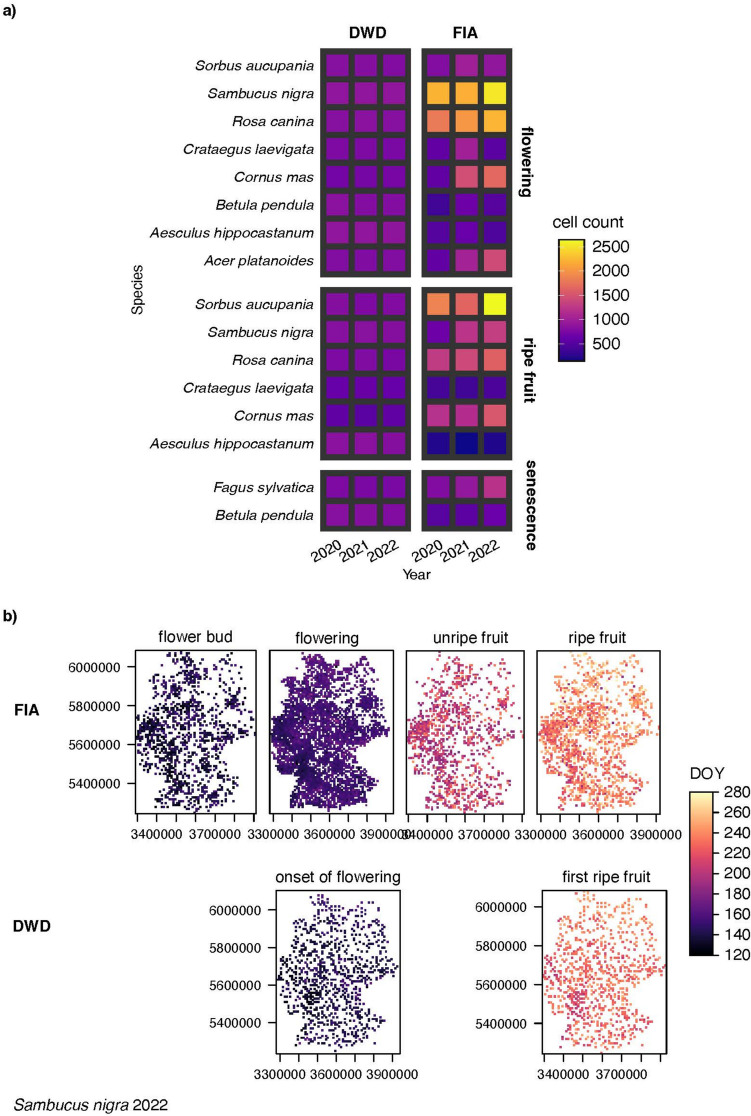
**a** Temporal coverage of phenological observations from systematic (DWD) and opportunistic (FIA) sources between 2020 and 2022, based on comparable phenostages: flowering, ripe fruit, and senescence. The color code refers to the number of gridcells with at least one observation of a phenostage in a given year. **b** Example for *Sambucus nigra* in 2022 at 10 × 10 km resolution in Germany. Grid cells with at least one observations are indicated by a color code expressing the day of year (DOY) when the observation was made: Upper panel: FIA-derived phenostages; lower panel displays corresponding DOY records from DWD for two observed stages

To compare the spatial distribution of opportunistic and systematic phenological data sets, we counted the number of 10 × 10 km grid cells containing FIA observation and compared them to the grid cells containing DWD observations. The DWD data exhibit consistent observation counts across years and species, reflecting their systematic and standardized nature. The number of cells containing observations from DWD and FIA is shown in Fig. 3a.

Notably, for 11 out of the 16 species-specific phenostages analyzed, the number of grid cells covered by FIA has shown an annual increase.

Figure 3b shows all phenological stages of *Sambucus nigra* across Germany in 2022, based on observations from both FIA and DWD data. Earlier phenological stages, such as flowering bud, are observed earlier in the year, whereas later stages, such as ripe fruit, occur later in the year. The spatial distribution of phenostages also reveals distinct patterns influenced by elevation and geomorphology. For instance, flowering in FIA data shows an earlier onset in low-elevation regions, such as the heat-favored Rhine River valley in western Germany, compared to higher-elevation regions where flowering occurs later.

Notably, FIA data for *Sambucus nigra* (Fig. 3b) provide two additional phenostages and show a broader spatial coverage compared to the systematic ones, emphasizing the potential of opportunistic observations to complement traditional monitoring data.

#### Differences in the timing of phenostages between DWD and FIA observations

**Fig. 4:**
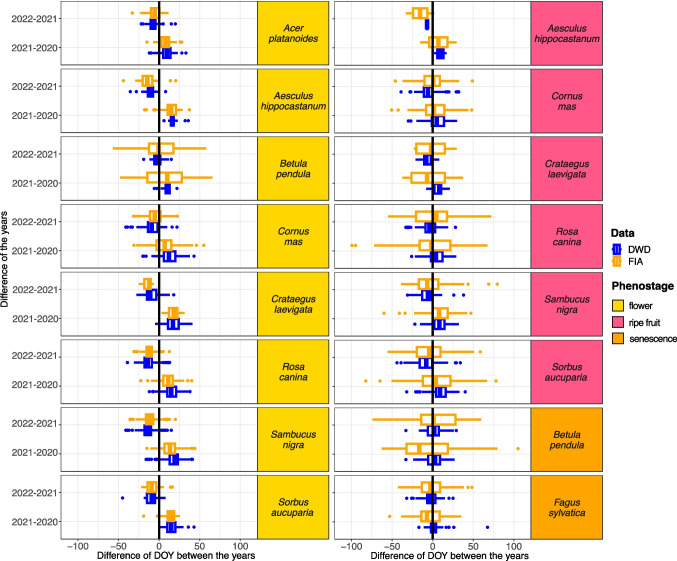
Interannual variability in phenostages within species, comparing opportunistic and systematic data. The boxplots display differences in the day of the year (DOY) for matching grid cells across different years, categorized by time intervals, species, and phenostages. Blue boxplots represent DWD, while orange boxplots correspond to observations from FIA

The distribution of differences between the same grid cells in consecutive years are similar in many cases, as shown in Fig. 4. The flowering stages of all considered species was observed later in 2021 than in 2020. In contrast, the difference between 2021 and 2022 is negative, indicating that flowering in 2022 occurred earlier than in 2021 across both data sets. In general, the distribution and dispersion of DOYs is very similar for the flowering stage, except for *Betula pendula*, where the DOYs differences show a markedly larger dispersion per grid cell. Also, for the ripe fruit stage of *Sorbus aucuparia*, *Rosa canina*, *Crataegus laevigata* and *Aesculus hippocastanaum*, the differences of the FIA-derived phenology estimates show a markedly larger variation than their DWD counterparts. Year-to-year differences become less pronounced as the phenological stage of the species advances. The ripe fruit stage exhibits less consistency compared to flowering. Among the six species, *Sambucus nigra* and *Sorbus aucuparia* show the highest agreement between the two data sets, with the median differences between years closely aligned. However, for the other four species, this pattern was less evident. Notably, *Cornus mas*, *Crataegus laevigata*, and *Rosa canina* display opposing trends between the two data sets, underscoring discrepancies in the timing of the ripe fruit. For the senescence, recorded for only two species, the DWD data exhibits no interannual differences, with median DOY differences remaining consistently close to zero. In contrast, the FIA data show similar consistency only between 2021 and 2022.

#### Pairwise mantel test

The pairwise Mantel correlations for each phenostage and year are presented in Table 2. The number of shared grid cells varies substantially between years and phenostages. For instance, grid cell counts for flowering range from 155 for *Cornus mas* in 2020 to 602 for *Sambucus nigra* in 2021. In general, almost all of the flowering stages in all years show highly significant mantel correlations, indicating that the differences between the grid cells of both data sets are based on similar spatial gradients. *Acer platanoides*, *Crataegus laevigata*, *Sorbus aucuparia*, *Sambucus nigra* and *Rosa canina* show consistently significant correlations across all three years. *Cornus mas* shows significant correlation in 2021 and 2022, but not in 2020, probably due to the low number of shared grid cells in that year. Similarly, *Aesculus hippocastanum* shows significant correlation for 2020 and 2021, but not 2022, in which the number of shared grid cells drops to only 136. *Betula pendula* is the only species that did not show any significant correlation between all three years in flowering. For ripe fruit, correlations are generally lower and more variable. Significant spatial correlations are observed for *Sambucus nigra* in 2022 (r M = 0.076) and for *Rosa canina* in 2021 (r M = 0.040), while other species show nonsignificant or negative correlations. Senescence displays modest correlations, with *Betula pendula* and *Fagus sylvatica* both showing significant correlations for at least one year. The results indicate that spatial patterns in phenostage timing captured by FIA align moderately well with those recorded by the systematic DWD network for certain species and phenostages. Notably, the flowering stage shows consistently stronger correlations than later stages, such as ripe fruit and senescence.

**Table 2:** Pairwise simple Mantel correlations of systematic phenological records by DWD and opportunistic estimates from FIA across the same grid cells with observations. rM refers to simple Mantel correlation and significant positive mantel correlations are denoted with stars: *p* < 0.05*, *p* < 0.01** *p* < 0.001***. Cell count denotes the the number of shared grid cells between DWD and FIA observations for each year. The species are ordered by the mean onset of their corresponding phenostage in the course of the year as reported by DWD

Species	Pheno-stage	Cell count	2020 *r* M	Cell count	2021 *r* M	Cell count	2022 *r* M
*Cornus mas*	flowering	155	0.053^*ns*^	372	0.135***	428	0.125***
*Acer platanoides*	flowering	183	0.108**	264	0.192***	363	0.192***
*Betula pendula*	flowering	101	−0.076^*ns*^	176	0.039^*ns*^	150	0.021^*ns*^
*Aesculus hippocastanum*	flowering	174	0.111*	203	0.119*	136	0.081^*ns*^
*Crataegus laevigata*	flowering	144	0.168***	250	0.136***	140	0.275***
*Sorbus aucuparia*	flowering	209	0.161***	280	0.181***	215	0.255***
*Sambucus nigra*	flowering	591	0.267***	602	0.211***	675	0.186***
*Rosa canina*	flowering	463	0.267***	542	0.181***	555	0.272***
*Sorbus aucuparia*	ripe fruit	493	0.005^*ns*^	383	−0.026^*ns*^	609	−0.029^*ns*^
*Cornus mas*	ripe fruit	241	0.051*	224	0.055^*ns*^	302	0.037^*ns*^
*Sambucus nigra*	ripe fruit	188	0.008^*ns*^	332	−0.022^*ns*^	333	0.076**
*Rosa canina*	ripe fruit	309	−0.002^*ns*^	341	0.007^*ns*^	367	0.004^*ns*^
*Crataegus laevigata*	ripe fruit	83	−0.069^*ns*^	87	0.040^*ns*^	92	−0.062^*ns*^
*Aesculus hippocastanum*	ripe fruit	66	0.035^*ns*^	41	−0.076^*ns*^	50	0.023^*ns*^
*Betula pendula*	senescence	152	0.060*	150	0.035^*ns*^	171	0.036^*ns*^
*Fagus sylvatica*	senescence	204	−0.030^*ns*^	235	0.088*	296	0.019^*ns*^

## Discussion

Our study shows that the automated phenostage annotation of plant images is possible with reasonable effort and a limited number of available training images. Such an approach enables harnessing secondary data from plant images which are, e.g., components of primary community science observations (Depauw et al. 2022; Pernat et al. 2024) and can be used to supplement and broaden available data sources for phenological research.

### Automated phenostage annotation

To our knowledge so far only two studies (Reeb et al. 2022; Dinnage et al. 2024) have successfully implemented automatic classification of phenological stages using iNaturalist observations.

A major challenge in using community science images lies in their high variability. Unlike standardized data sets, such as automated repeated photographs or herbarium specimens, community science images are highly diverse. Photos may be taken from various perspectives, ranging from close-ups of a tree blossom to full-frame images of an entire tree. This variability makes automated phenological stage classification significantly more complex compared to data from experimental or standardized settings (López-Guillén et al. 2024).

Nevertheless, developing an automatic image recognition system from scratch using CNNs is both computationally expensive and time-intensive (Zhao et al. 2024). CNNs typically require large, labeled data sets to achieve reliable performance (Krizhevsky et al. 2017; Zhao et al. 2024). The extensive manual annotation required to train such models is a significant bottleneck for their practical application. For example, Reeb et al. (2022) utilized more than 12,000 manually annotated images of *Alliaria petiolata* to classify four phenological stages, achieving an overall accuracy of 86.4%. They used a CNN pre-trained on the ImageNet data set (Deng et al. 2009), containing over one million images across 1,000 object classes. While any kind of pre-training is beneficial for a classification task (Pan 2010), networks that have been pre-trained on similar problems have been proven useful in other fine-grained classification tasks (Hodač et al. 2024; Rzanny et al. 2022; Valan et al. 2019).

Overall, our study presents a scalable and efficient approach to automate phenological stage classification. With only minimal manual annotation efforts (2,890 training images across nine species), we achieved a robust classification performance. Moreover, the computational demands for training our SVM model were manageable on a standard laptop. This demonstrates that, once an appropriate pre-trained network is available, automated phenological stage annotation can be effectively applied to individual species. This makes our method both accessible and practical for large-scale phenological research.

### Opportunistic plant observation data for phenological research

To validate the reliability of automated annotation for thousands of plant images and the derived phenological data for research purposes, we compared the opportunistic FIA data with the systematically recorded DWD data. Specifically, we examined if both data sets exhibit corresponding interannual and spatial variations in a year for the timing of certain phenological events. Both interannual and spatial variations of phenological events are crucial metrics and frequently incorporated into phenological models (Primack et al. 2023).

Since the similarity in flowering stages in both data sets was quite high, it indicates that the flowering data from a plant identification app can be reliably compared to systematic phenological data. The reason lies in conspicuous flowers that are frequently photographed by app users, and short flowering periods, resulting in a distinct observation curve. This is consistent with a previous study, which also showed that opportunistic data for species with noticeable flowers are well-suited for phenological research (Katal et al. 2022).

An exception is the birch tree (*Betula pendula*), where the observed flowering shows greater interannual variation and different spatial patterns in the FIA than in the DWD data. Birch flowers are wind-pollinated and lack conspicuous petals, making it difficult to recognize the beginning of flowering in the community science data. Male and female catkins develop over several months, with females gradually transitioning into the fruiting stage (Ranta et al. 2008). The full flowering stage is not significantly more visually distinct than the unripe or withered stage. While phenological observers can determine flowering onset by the opening of male catkins, community science images often capture unripe, dormant, or withered ones, which cannot be visually distinguished. This leads to an extended observation window, increased variation in DOY estimates, and reduced reliability for phenological monitoring. To improve accuracy, future efforts should refine this phenological stage identification.

Compared to the flowering stages, larger discrepancies were observed in the fruiting stage between the FIA and the DWD dataset. This is likely due to the longer duration of fruiting and its gradual transition from flowering, which makes it more difficult to determine its exact time. The fruiting for nearly all of the species we investigated spanned two to three months, meaning that fruits remain on the plant for an extended period, for example in *Crataegus laevigata* fruits can ripen from June to November (Thomas et al. 2021; Klymenko & Ilyinska 2023). Consequently, the time during which the fruits catch the observer’s attention becomes much more variable. Therefore, species such as *Sambucus nigra* (Atkinson & Atkinson 2002), with berry- like fruits that ripen and then quickly drop from the shrub, exhibit a phenological pattern that matches the DWD data more closely. In contrast, *Rosa canina*, whose fruits remain on the plant for two to three months until consumed by birds, shows greater variability in its fruiting stage.

Senescence also shows considerable variation, primarily because in systematic monitoring, a precise definition is used: approximately 50% of the leaves must have developed autumn coloration (Wetterdienst (DWD) 2015). In contrast, FIA observations often capture individual leaves, making it impossible to know how many leaves have actually changed color on the entire tree. As a result, the observations can include leaves from the very beginning of the senescence phase (with only a few leaves starting to discolor) all the way to the end of autumn when colorful leaves fall to the ground. This leads to a prolonged documentation of this stage compared to the systematic monitoring.

### Fine-grained phenological monitoring

Our results suggest that automated phenostage annotation of images may become important to mitigate the consequences of the loss of phenological observers (Yuan et al. 2021). Recognizing several phenostages on images offers the opportunity for a more fine-grained phenological monitoring for individual species than what has previously been achieved through the systematic monitoring. Here we could facilitate 14 additional phenological stages for the selected species that are currently not considered in the monitoring scheme of the DWD. As a consequence, we could not directly compare them with the systematic monitoring data. However, the interannual patterns for these additional stages reveal that early ones, such as flowering bud, precede the flowering stage, with 2021 showing a later onset compared to 2020 and 2022 (Figure S5). Fine-grained phenological monitoring offers significant ecological, health, and economic benefits. Ecologically, investigating the timing of senescence in species such as *Fagus sylvatica* and *Acer platanoides* is critical for understanding ecosystem functioning, as senescence plays a key role in regulating carbon and nutrient cycling through processes like leaf litter production and nutrient resorption (Panchen et al. 2015; Calinger and Curtis 2023). Moreover, monitoring flowering phenology by species like *Sambucus nigra*, *Cornus mas*, and *Rosa Canina* provides essential insights into plant–pollinator interactions, which are vital for maintaining biodiversity and ecosystem resilience. Shifts in flowering times can lead to phenological mismatches between plants and their pollinators, disrupting mutualistic networks and potentially reducing reproductive success and pollinator survival (Kudo and Ida 2013; Mach and Potter 2018). From a health perspective, monitoring the flowering buds or flowering stages of highly allergenic species like *Betula pendula* enables more accurate predictions of pollen release, facilitating the development of early warning systems and reducing health risks (Scheifinger et al. 2013). Economically, fine-grained monitoring of stages such as unripe fruit and ripe fruit can improve agricultural practices by helping farmers make more informed decisions regarding the timing of pesticide applications and harvests (Taylor and Browning 2022; Hufkens et al. 2019).

### Future perspective and challenges

Apps like Flora Incognita are primarily designed for plant species identification. Unlike phenological observers, who systematically monitor specific plants over time (Kaspar et al. 2015), plant identification app users document plants for diverse reasons, leading to known biases (Carlen et al. 2024; Geurts et al. 2023; Knape et al. 2022). These biases result in observations being more frequent in densely populated areas, during good weather, on weekends, public holidays, and near roads. However, similar biases, albeit to a lesser extent, also affect various community science datasets, including the phenological records of the DWD (Courter et al. 2013). Figure 3a compares the spatial coverage of observations per phenostage between DWD and FIA. While FIA data provide broader coverage in urban areas, records are sparser in less populated regions. As the number of systematic observers declines, plant identification apps generate an increasing volume of opportunistic observations (Table S2), offering valuable data on plant occurrences.

While our analysis was restricted to species monitored by the DWD, thousands of observations exist for many other plant species (Rzanny et al. 2024b), which could be annotated similarly. This approach would be particularly beneficial for herbaceous species, which are rarely monitored (Nordt et al. 2021). Furthermore, it can be readily extended to images from large online databases (Jarić et al. 2020; Klinger et al. 2023; August et al. 2020), expanding its applicability beyond the datasets examined here.

To conclude, plant identification apps are used by millions of people, generating an unprecedented and ever-growing digital collection of observations with timestamps, locations, and images from around the world. This study presents a straightforward approach to utilizing this vast and diverse dataset for phenological research. While phenological records from public authorities are typically confined to single countries and follow strict protocols, image data from plant identification apps transcend national borders and are collected without predefined guidelines. By harnessing this extensive biological information and implementing strategies such as tailored gamification to engage and motivate users, the volume of data collected for specific research tasks could be further expanded (Balmford et al. 2002). With thoughtful implementation and methodological refinements, opportunistic plant observations from identification apps have the potential to significantly enhance phenological monitoring, offering new opportunities to track ecological changes at an unprecedented scale.

## Supplementary Information

Below is the link to the electronic supplementary material.

Supplementary file1 (JPG 280 KB) Temporal distribution of Flora Incognita observations between 2020 to 2022 in Germany

Supplementary file2 (JPG 432 KB) Confusion matrix of the trained SVM model. The confusion matrix reveals that misclassifications were primarily between phenostages within the same species. For instance, ”*flowering bud*” images were misclassified as the *”flower”* stage, the *”unripe fruit*” stage, or the ”*vegetative*” stage within the same species. ”*vegetative*” stages were predominantly misclassified as ”*senescence*” also within the same species. Additional intraspecific misclassifications involved the *”unripe fruit”*, *”ripe fruit”,* *”flower”* and *”senescence”* stages. The only instance of confusion of interspecies confusion occurred when a *”vegetative”* image of *Cornus mas *was incorrectly classified as a *”vegetative” *stage of *Crataegus laevigata*

Supplementary file3 (JPG 274 KB) Performance of the SVM model across different phenostages. The F1 score, ranging from 0 to 1, evaluates the classification accuracy for each phenostage across all species, with values closer to 1 indicating higher model performance

Supplementary file4 (JPG 329 KB) Proportion of *Flora Incognita *observations per phenostage for each species after automated phenostage annotation

Supplementary file5 (JPG 403 KB) Additional phenostages in the opportunistic plant observation data via Flora Incognita (FIA) which are not monitored by the German Meteorological Service (DWD). The boxplot illustrates the median DOY for each phenostage, while ”Diff” represents the difference in median DOY for the given phenostage compared to the previous year

Supplementary file6 (DOCX 313 KB)

## Data Availability

The code for training the SVM model, annotated data from iNaturalist and FIA, extracted image features, and 10 × 10 gridded maps for all species, years and phenostages will be made publicly available on the *Zenodo* repository 10.1007/s00484-025-02972-x.
