# Supplementary figures and images for: Expanding phenological insights: automated phenostage annotation with community science plant images
Source: Int J Biometeorol. 2025 Jul 4;69(9):2353–67. doi: 10.1007/s00484-025-02972-x (PMC12479636; doi:10.1007/s00484-025-02972-x)

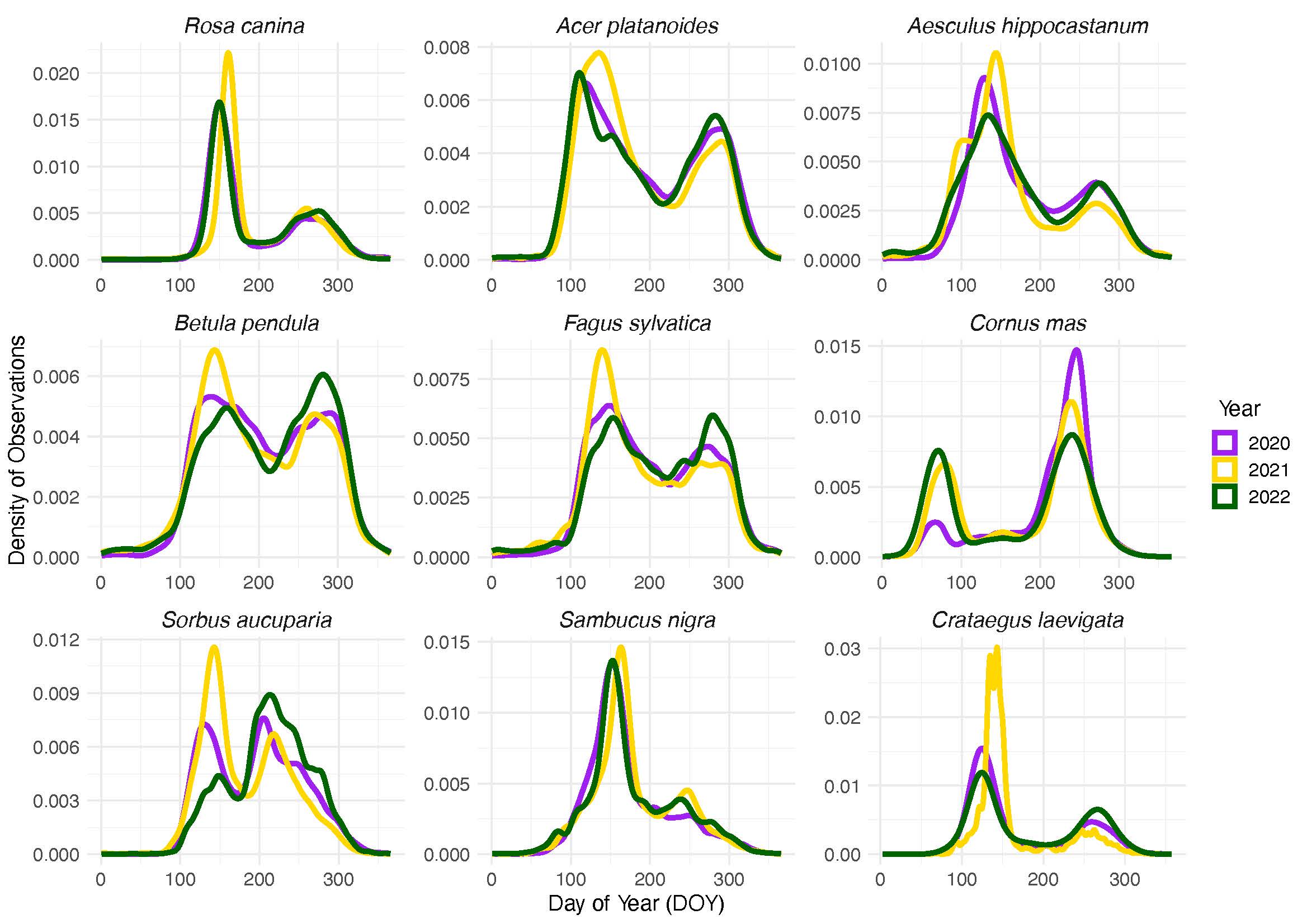

Supplement: Supplementary file 1 — Supplementary file1 (JPG 280 KB) Temporal distribution of Flora Incognita observations between 2020 to 2022 in Germany [file 484_2025_2972_MOESM1_ESM.jpg]

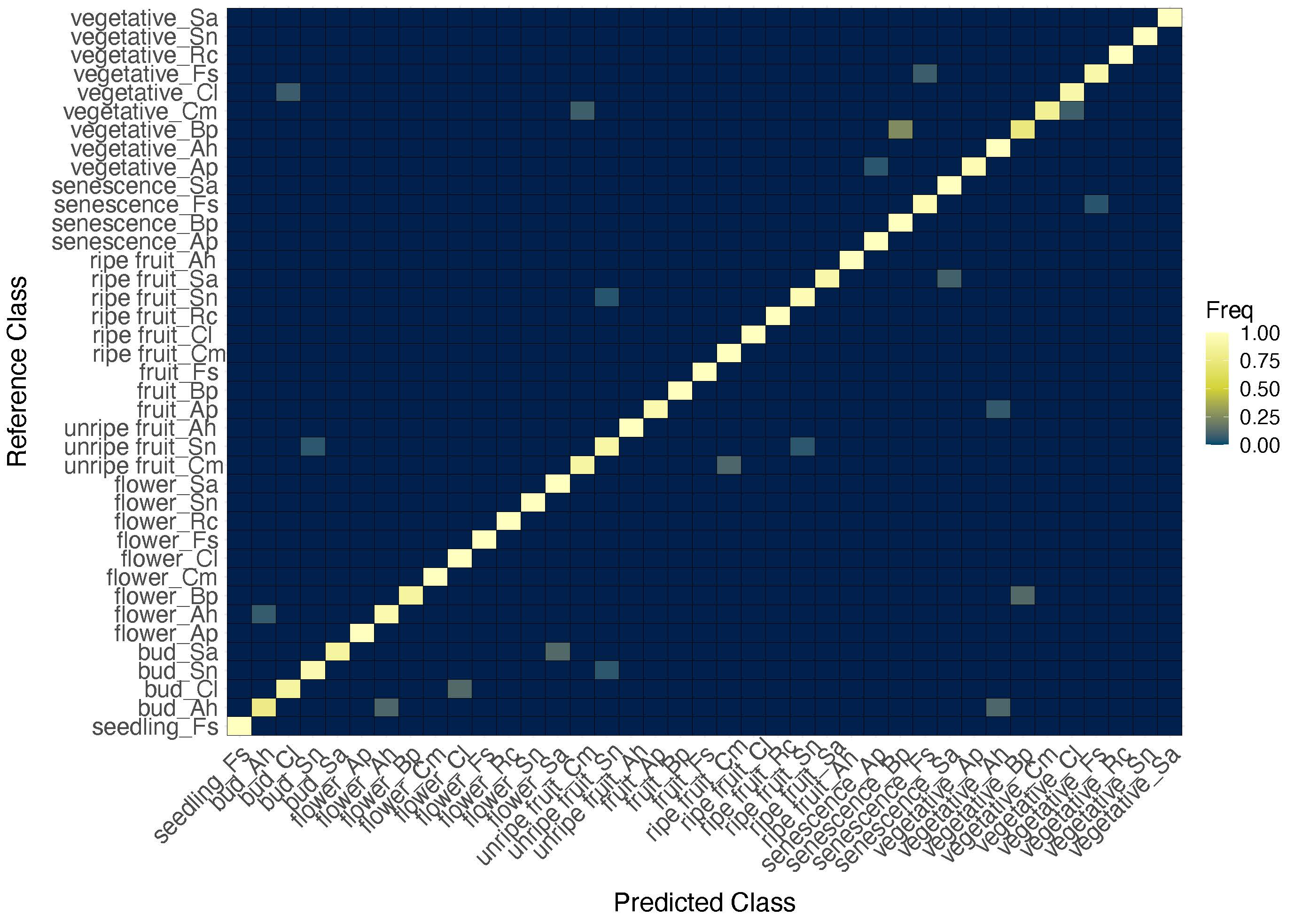

Supplement: Supplementary file 2 — Supplementary file2 (JPG 432 KB) Confusion matrix of the trained SVM model. The confusion matrix reveals that misclassifications were primarily between phenostages within the same species. For instance, ”flowering bud” images were misclassified as the ”flower” stage, the ”unripe fruit” stage, or the ”vegetative” stage within the same species. ”vegetative” stages were predominantly misclassified as ”senescence” also within the same species. Additional intraspecific misclassifications involved the ”unripe fruit”, ”ripe fruit”, ”flower” and ”senescence” stages. The only instance of confusion of interspecies confusion occurred when a ”vegetative” image of Cornus mas was incorrectly classified as a ”vegetative” stage of Crataegus laevigata [file 484_2025_2972_MOESM2_ESM.jpg]

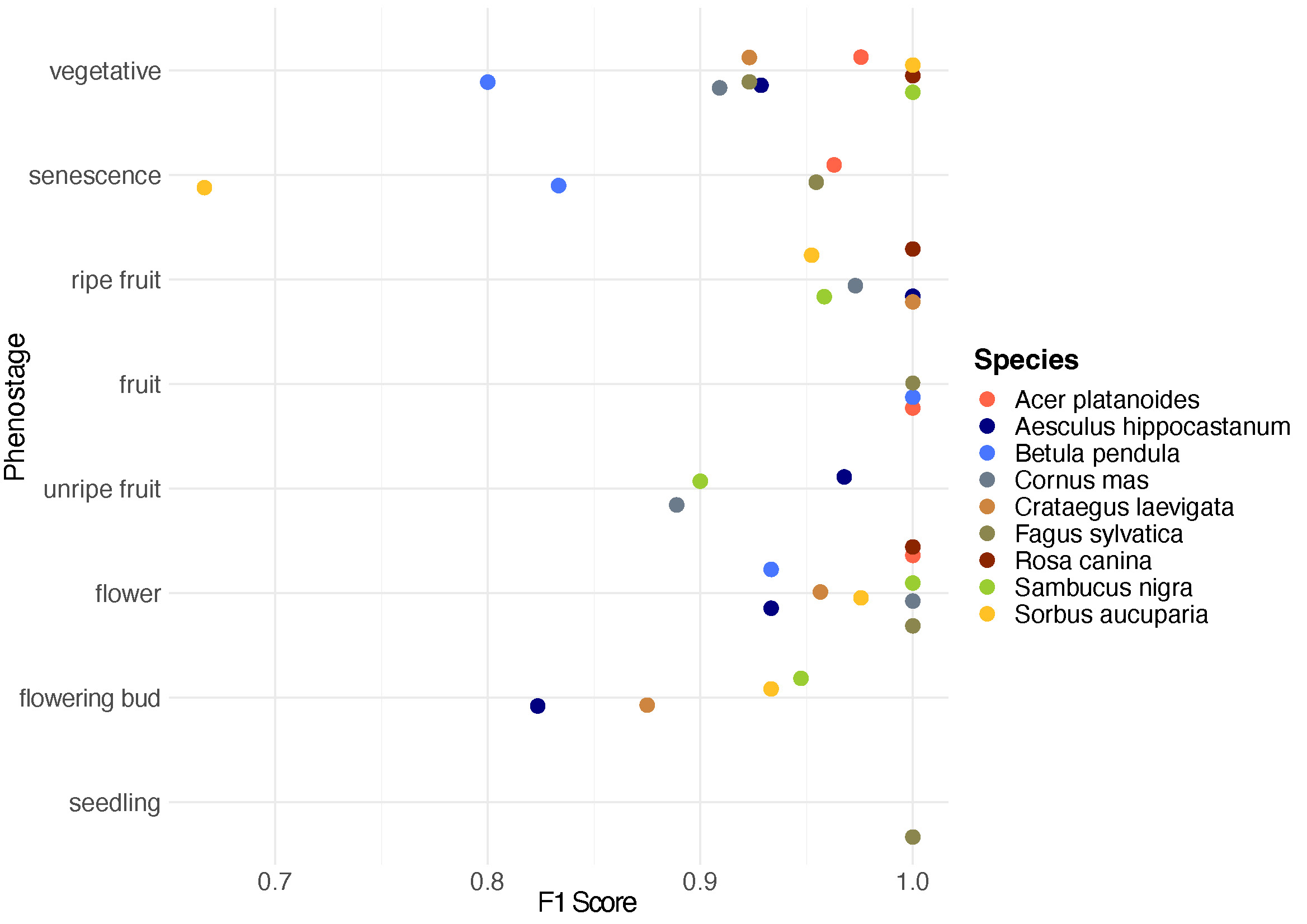

Supplement: Supplementary file 3 — Supplementary file3 (JPG 274 KB) Performance of the SVM model across different phenostages. The F1 score, ranging from 0 to 1, evaluates the classification accuracy for each phenostage across all species, with values closer to 1 indicating higher model performance [file 484_2025_2972_MOESM3_ESM.jpg]

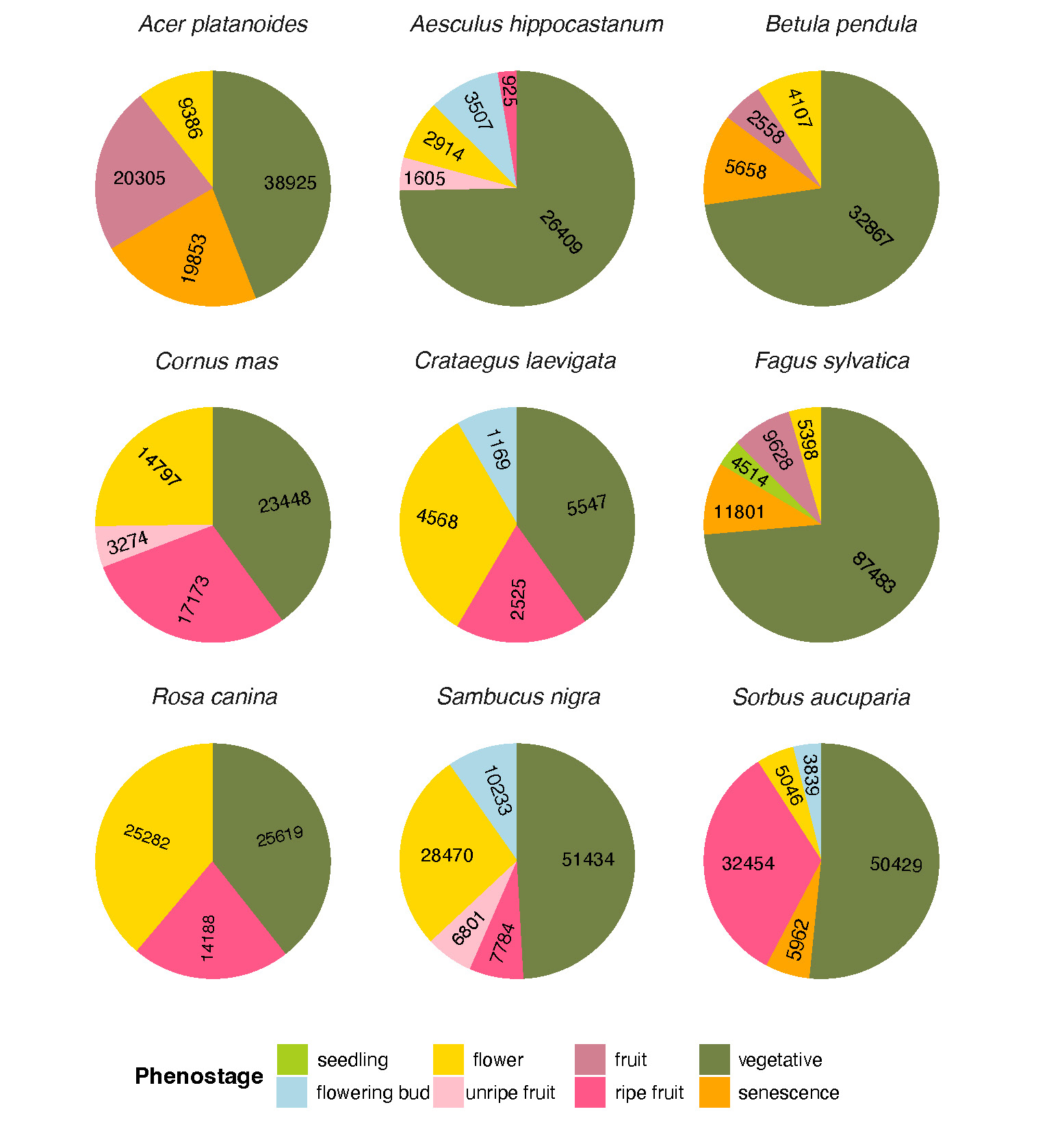

Supplement: Supplementary file 4 — Supplementary file4 (JPG 329 KB) Proportion of Flora Incognita observations per phenostage for each species after automated phenostage annotation [file 484_2025_2972_MOESM4_ESM.jpg]

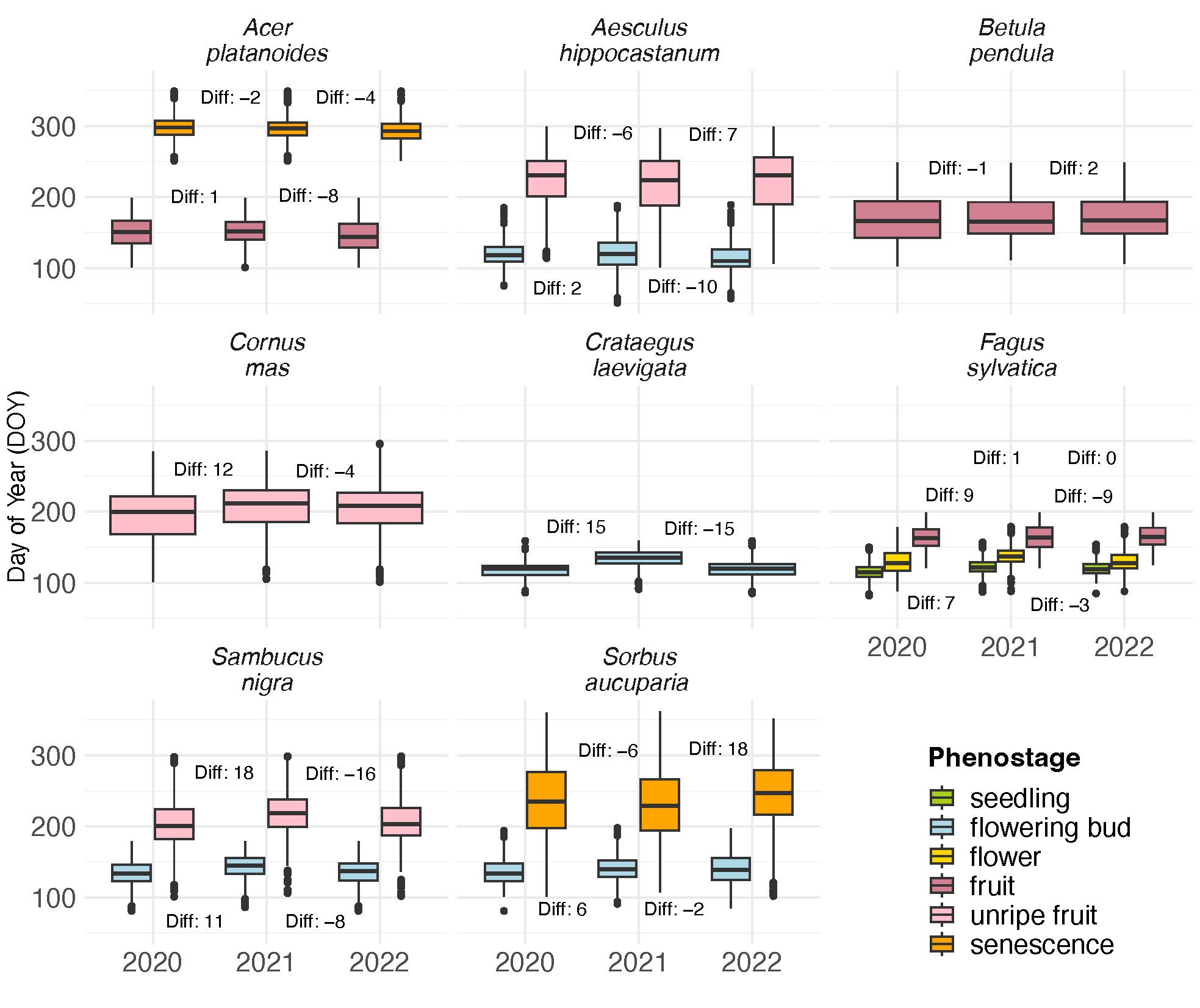

Supplement: Supplementary file 5 — Supplementary file5 (JPG 403 KB) Additional phenostages in the opportunistic plant observation data via Flora Incognita (FIA) which are not monitored by the German Meteorological Service (DWD). The boxplot illustrates the median DOY for each phenostage, while ”Diff” represents the difference in median DOY for the given phenostage compared to the previous year [file 484_2025_2972_MOESM5_ESM.jpg]
