# Supplementary material for: Expanding phenological insights: automated phenostage annotation with community science plant images
Source: Int J Biometeorol. 2025 Jul 4;69(9):2353–67. doi: 10.1007/s00484-025-02972-x (PMC12479636; doi:10.1007/s00484-025-02972-x)
Supplement: Supplementary file 6 — Supplementary file6 (DOCX 313 KB) [file 484_2025_2972_MOESM6_ESM.docx]

**Table S1**: Overview of the annotated phenostages per species, including examples of the corresponding images from *iNaturalist*. The numbers represent the number of annotated images per phenostage and species.


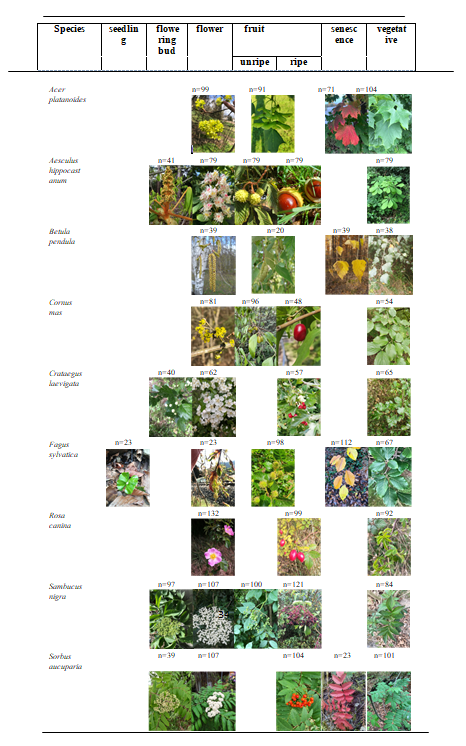


**Table S2**: Number of *Flora Incognita* observations per species, along with the number of observations and 10 × 10 km grid cells after automated phenostage annotation and data cleaning for each year.

| **Species** | **Obs.** | **Phenostage** | **Obs. before**  **cleaning** | **Obs. after**  **cleaning** | **2020**  **Obs. Cells** | | **2021**  **Obs. Cells** | | **2022**  **Obs. Cells** | |
| --- | --- | --- | --- | --- | --- | --- | --- | --- | --- | --- |
|  |  | flower | 9386 | 9131 | 1222 | 593 | 3203 | 1037 | 4706 | 1422 |
| *Acer*  *platanoides* | 88,469 | fruit  senescence | 20305  19853 | 10155  13897 | 2768  3576 | 1067  1095 | 3934  4056 | 1360  1200 | 3453  6265 | 1269  1253 |
|  |  | vegetative | 38925 | - | - | - | - | - | - | - |
|  |  | flowering bud | 3507 | 3143 | 734 | 476 | 1448 | 775 | 961 | 558 |
| *Aesculus hippocastanum* | 35,360 | flower  unripe fruit ripe fruit | 2914  1605  925 | 2631  1482  746 | 854  551  203 | 498  366  148 | 1048  361  174 | 634  268  130 | 729  570  369 | 458  375  187 |
|  |  | vegetative | 26409 | - | - | - | - | - | - | - |
|  |  | flower | 4107 | 2870 | 599 | 367 | 1361 | 662 | 910 | 507 |
| *Betula*  *pendula* | 45,190 | fruit  senescence | 2558  5658 | 2077  3759 | 834  1032 | 483  520 | 617  1112 | 362  550 | 626  1615 | 379  649 |
|  |  | vegetative | 32867 | - | - | - | - | - | - | - |
|  |  | flower | 14797 | 14628 | 1341 | 587 | 5623 | 1449 | 7664 | 1672 |
| *Cornus mas* | 58,692 | unripe fruit  ripe fruit | 3274  17173 | 3185  16299 | 1009  4556 | 494  1201 | 998  5071 | 464  1163 | 1178  6675 | 595  1531 |
|  |  | vegetative | 23448 | - | - | - | - | - | - | - |
|  |  | flowering bud | 1169 | 1128 | 225 | 187 | 622 | 431 | 281 | 217 |
| *Crataegus*  *laevigata* | 13,809 | flower  ripe fruit | 4568  2525 | 4142  2168 | 892  666 | 590  402 | 2230  584 | 1014  366 | 1020  918 | 542  442 |
|  |  | vegetative | 5547 | - | - | - | - | - | - | - |
|  |  | seedling | 4514 | 4176 | 522 | 227 | 3294 | 1085 | 360 | 124 |
|  | 118,824 | flower | 5398 | 3893 | 822 | 506 | 1569 | 798 | 1502 | 711 |
| *Fagus sylvatica* |  | fruit | 9628 | 4752 | 2007 | 947 | 744 | 463 | 2001 | 866 |
|  |  | senescence | 11801 | 7926 | 1805 | 785 | 2131 | 912 | 3990 | 1206 |
|  |  | vegetative | 87483 | - | - | - | - | - | - | - |
|  |  | flower | 25282 | 24472 | 7798 | 1806 | 8404 | 2025 | 8270 | 2234 |
| *Rosa canina* | 65,089 | ripe fruit | 14188 | 13520 | 3879 | 1288 | 4460 | 1410 | 5181 | 1611 |
|  |  | vegetative | 25619 | - | - | - | - | - | - | - |
|  |  | flowering bud | 10233 | 8768 | 2495 | 1225 | 3510 | 1471 | 2763 | 1362 |
| *Sambucus nigra* | 104,722 | flower  unripe fruit ripe fruit | 28470  6801  7784 | 27678  6587  6472 | 8861  1778  1270 | 2215  976  725 | 8743  2152  2478 | 2190  1156  1290 | 10074  2657  2724 | 2532  1342  1373 |
|  |  | vegetative | 51434 | - | - | - | - | - | - | - |
|  |  | flowering bud | 3839 | 3703 | 1012 | 673 | 1174 | 742 | 1517 | 881 |
| *Sorbus aucuparia* | 97,730 | flower  ripe fruit senescence | 5046  32454  5962 | 4623  29669  5930 | 1165  5067  1238 | 790  1876  785 | 1826  4291  1231 | 1001  1647  805 | 1632  20311  3461 | 898  2645  1398 |
|  |  | vegetative | 50429 | - | - | - | - | - | - | - |

**Table S3**: Number of observations and 10 × 10 km grid cells with recorded phenology data from the German Meteorological Service (DWD) between January 1, 2020, and December 31, 2022.

| **Species** | **Phenostage** | **Obs.** | **2020**  **Cells** | **Obs.** | **2021**  **Cells** | **Obs.** | **2022**  **Cells** |
| --- | --- | --- | --- | --- | --- | --- | --- |
| *Acer platanoides* | flowering onset | 836 | 788 | 816 | 770 | 838 | 788 |
| *Aesculus hippocastanum* | flowering onset | 954 | 887 | 958 | 888 | 944 | 875 |
|  | first ripe fruits | 905 | 841 | 904 | 837 | 880 | 818 |
| *Betula pendula* | flowering onset | 902 | 837 | 850 | 792 | 855 | 798 |
|  | leaf senescence | 887 | 819 | 872 | 811 | 844 | 784 |
| *Cornus mas* | flowering onset | 751 | 703 | 767 | 716 | 776 | 724 |
|  | first ripe fruits | 613 | 579 | 570 | 541 | 604 | 574 |
| *Crataegus laevigata* | flowering onset | 813 | 764 | 813 | 762 | 784 | 738 |
|  | first ripe fruits | 707 | 626 | 674 | 637 | 661 | 626 |
| *Fagus sylvatica* | leaf senescence | 822 | 762 | 799 | 749 | 790 | 739 |
| *Rosa canina* | flowering onset | 879 | 823 | 907 | 842 | 885 | 821 |
|  | first ripe fruits | 809 | 761 | 821 | 770 | 790 | 736 |
| *Sambucus nigra* | flowering onset | 963 | 887 | 965 | 891 | 968 | 889 |
|  | first ripe fruits | 885 | 825 | 899 | 834 | 884 | 818 |
| *Sorbus aucuparia* | flowering onset | 883 | 821 | 881 | 815 | 848 | 786 |
|  | first ripe fruit | 872 | 813 | 820 | 765 | 844 | 785 |
